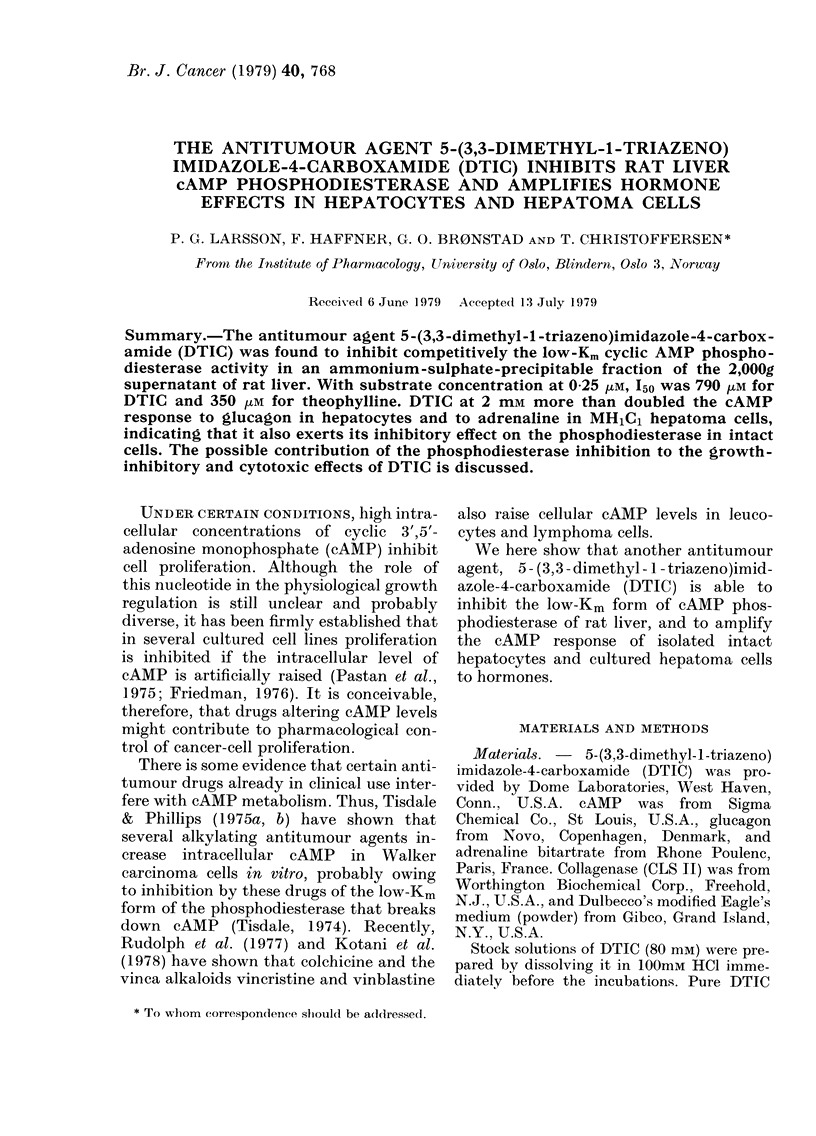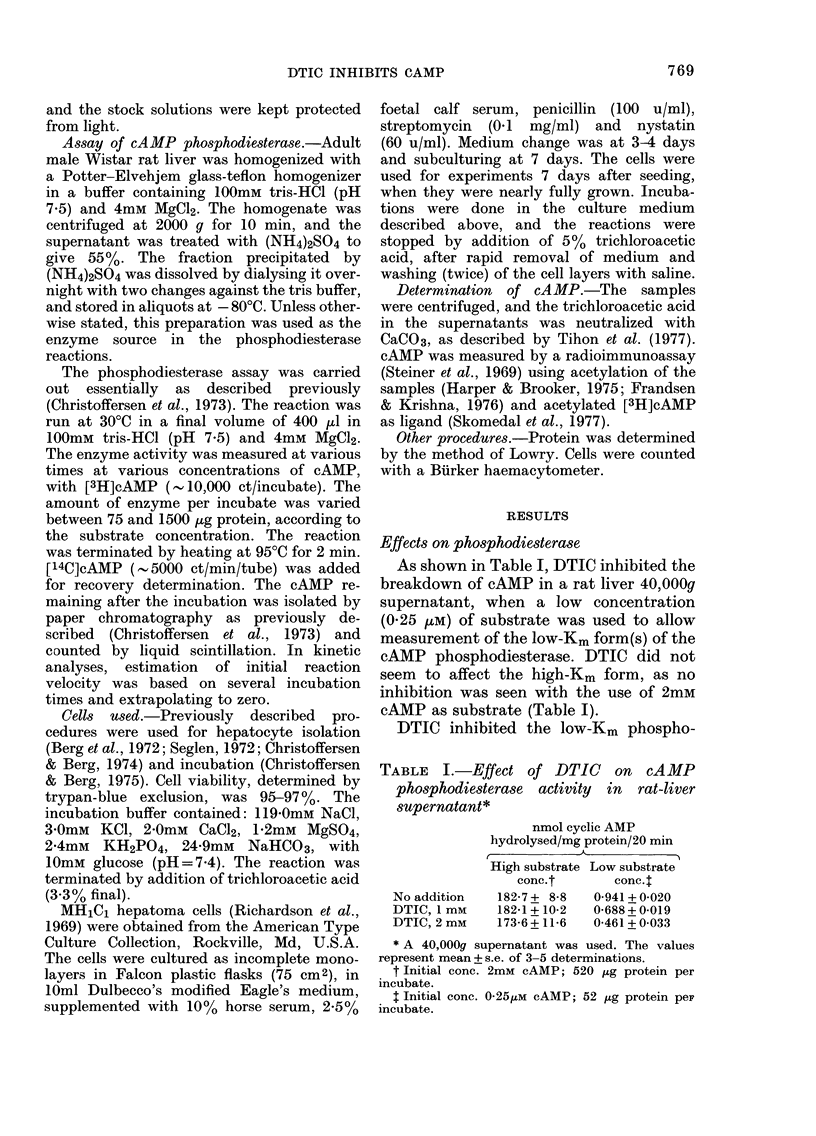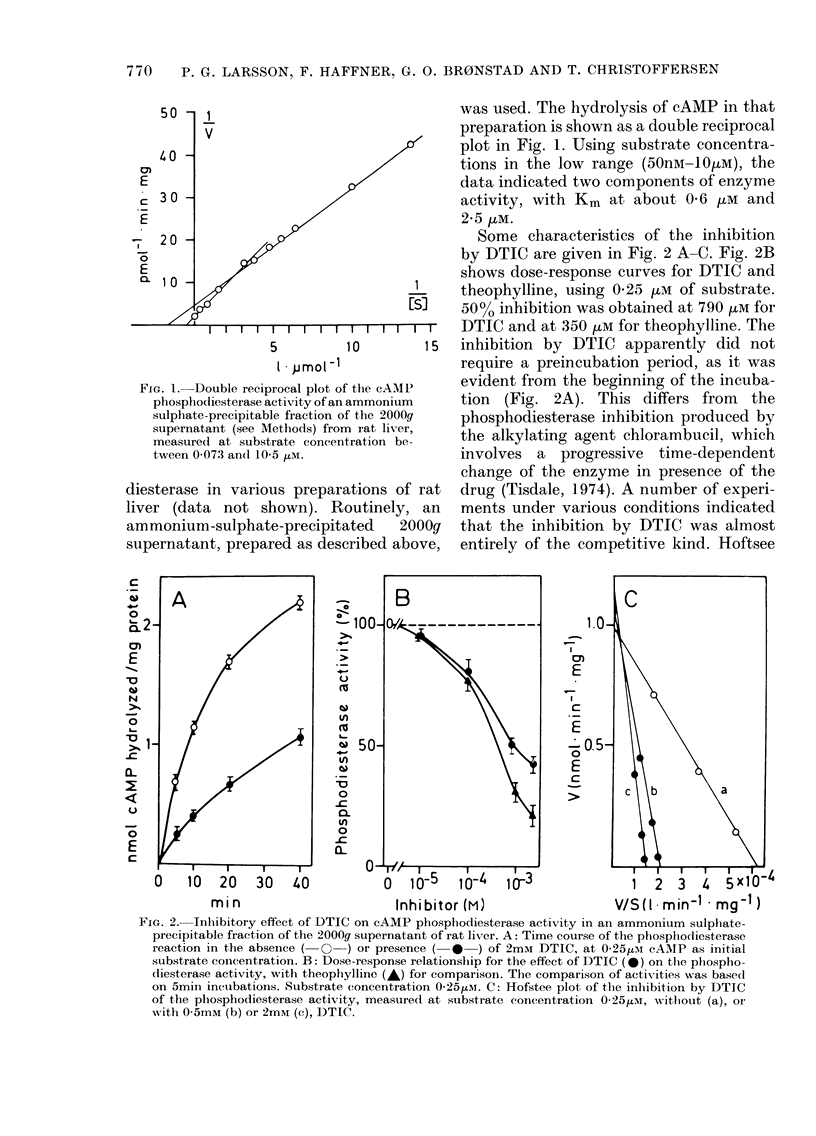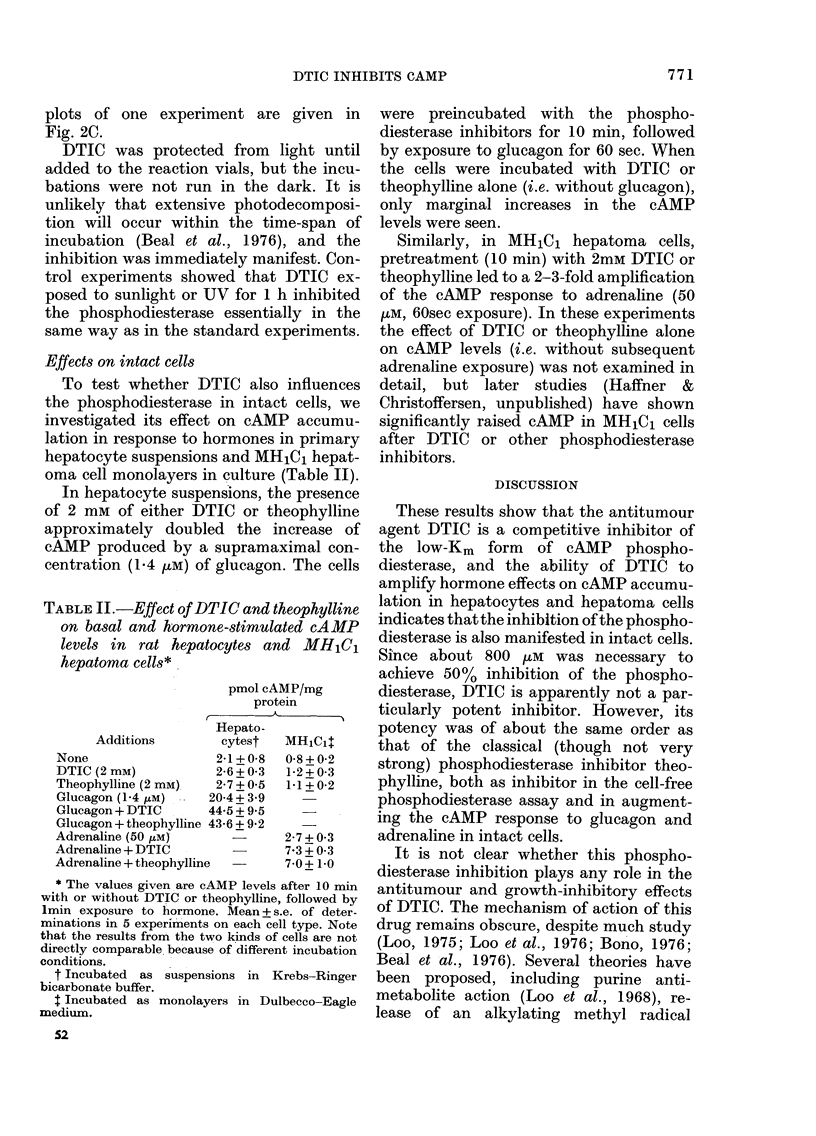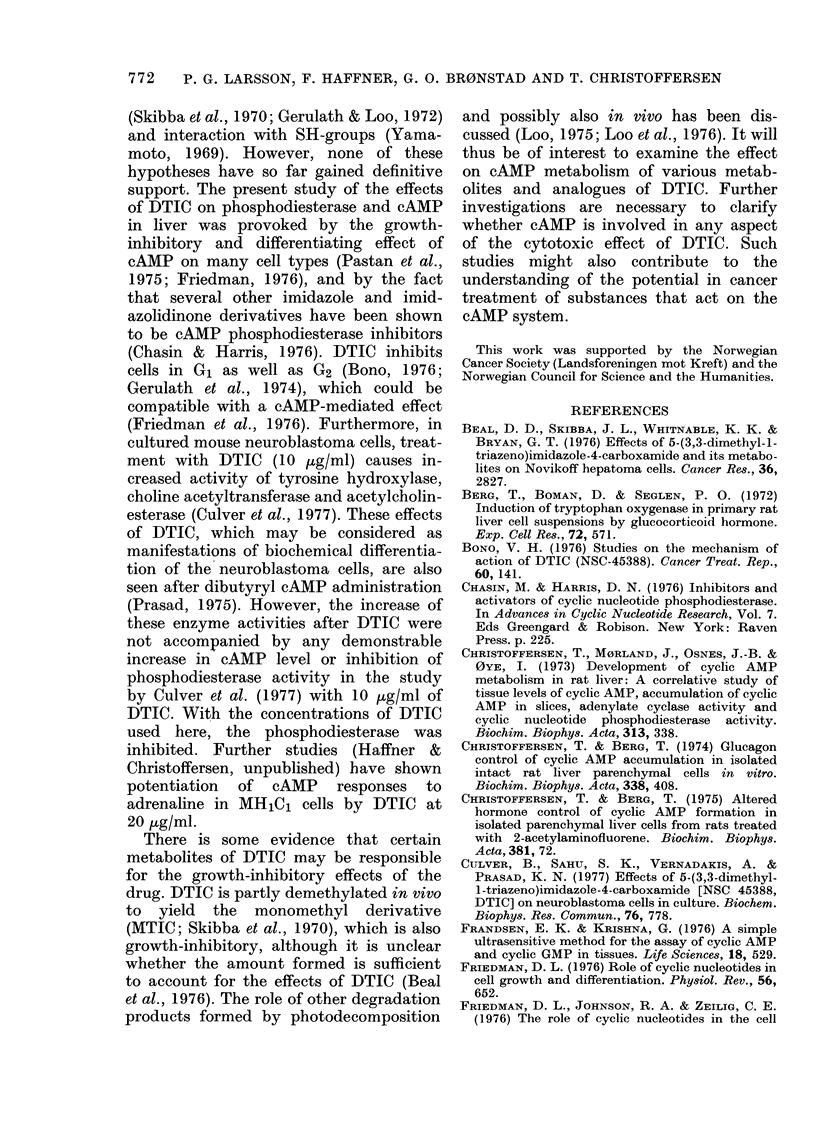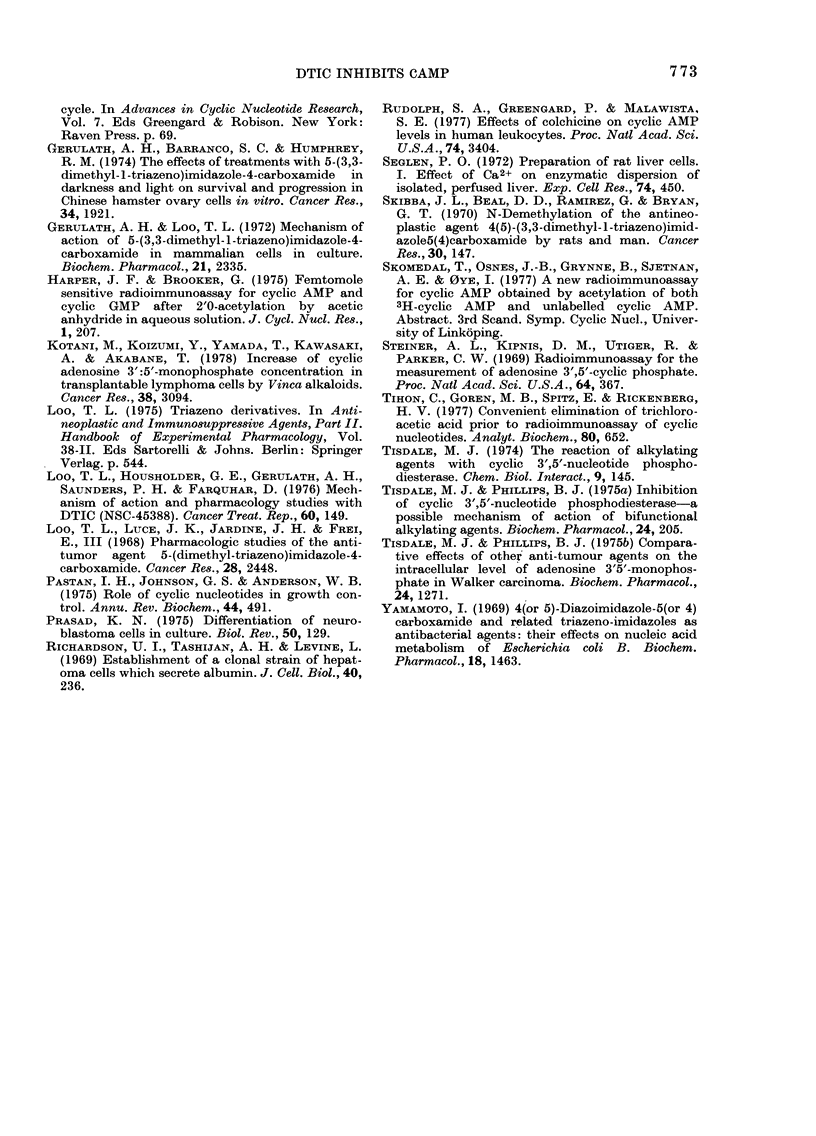# The antitumour agent 5-(3,3-dimethyl-1-triazeno) imidazole-4-carboxamide (DTIC) inhibits rat liver cAMP phosphodiesterase and amplifies hormone effects in hepatocytes and hepatoma cells.

**DOI:** 10.1038/bjc.1979.259

**Published:** 1979-11

**Authors:** P. G. Larsson, F. Haffner, G. O. Brłnstad, T. Christoffersen

## Abstract

The antitumour agent 5-(3,3-dimethyl-1-triazeno)imidazole-4-carboxamide (DTIC) was found to inhibit competitively the low-Km cyclic AMP phosphodiesterase activity in an ammonium-sulphate-precipitable fraction of the 2,000g supernatant of rat liver. With substrate concentration at 0.25 microM, I50 was 790 microM for DTIC and 350 microM for theophylline. DTIC at 2 mM more than doubled the cAMP response to glucagon in hepatocytes and to adrenaline in MH1C1 hepatoma cells, indicating that it also exerts its inhibitory effect on the phosphodiesterase in intact cells. The possible contribution of the phosphodiesterase inhibition to the growth-inhibitory and cytotoxic effects of DTIC is discussed.


*Br. J. Cancer* (1979) **40**, 768

### THE ANTITUMOUR AGENT 5-(3,3-DIMETHYL-1-TRIAZENO) IMIDAZOLE-4-CARBOXAMIDE (DTIC) INHIBITS RAT LIVER cAMP PHOSPHODIESTERASE AND AMPLIFIES HORMONE EFFECTS IN HEPATOCYTES AND HEPATOMA CELLS

P. G. LARSSON, F. HAFFNER, G. O. BRØNSTAD AND T. CHRISTOFFERSEN*

*From the Institute of Pharmacology, University of Oslo, Blindern, Oslo 3, Norway*

Received 6 June 1979 Accepted 13 July 1979

**Summary.—The antitumour agent 5-(3,3-dimethyl-1-triazeno)imidazole-4-carboxamide (DTIC) was found to inhibit competitively the low-$K_m$ cyclic AMP phosphodiesterase activity in an ammonium-sulphate-precipitable fraction of the 2,000*g* supernatant of rat liver. With substrate concentration at 0·25 $\mu$M, $I_{50}$ was 790 $\mu$M for DTIC and 350 $\mu$M for theophylline. DTIC at 2 mM more than doubled the cAMP response to glucagon in hepatocytes and to adrenaline in $MH_1C_1$ hepatoma cells, indicating that it also exerts its inhibitory effect on the phosphodiesterase in intact cells. The possible contribution of the phosphodiesterase inhibition to the growth-inhibitory and cytotoxic effects of DTIC is discussed.**

UNDER CERTAIN CONDITIONS, high intracellular concentrations of cyclic 3′,5′-adenosine monophosphate (cAMP) inhibit cell proliferation. Although the role of this nucleotide in the physiological growth regulation is still unclear and probably diverse, it has been firmly established that in several cultured cell lines proliferation is inhibited if the intracellular level of cAMP is artificially raised (Pastan *et al.*, 1975; Friedman, 1976). It is conceivable, therefore, that drugs altering cAMP levels might contribute to pharmacological control of cancer-cell proliferation.

There is some evidence that certain antitumour drugs already in clinical use interfere with cAMP metabolism. Thus, Tisdale & Phillips (1975*a*, *b*) have shown that several alkylating antitumour agents increase intracellular cAMP in Walker carcinoma cells *in vitro*, probably owing to inhibition by these drugs of the low-$K_m$ form of the phosphodiesterase that breaks down cAMP (Tisdale, 1974). Recently, Rudolph *et al.* (1977) and Kotani *et al.* (1978) have shown that colchicine and the vinca alkaloids vincristine and vinblastine also raise cellular cAMP levels in leucocytes and lymphoma cells.

We here show that another antitumour agent, 5-(3,3-dimethyl-1-triazeno)imidazole-4-carboxamide (DTIC) is able to inhibit the low-$K_m$ form of cAMP phosphodiesterase of rat liver, and to amplify the cAMP response of isolated intact hepatocytes and cultured hepatoma cells to hormones.

### MATERIALS AND METHODS

*Materials.* — 5-(3,3-dimethyl-1-triazeno) imidazole-4-carboxamide (DTIC) was provided by Dome Laboratories, West Haven, Conn., U.S.A. cAMP was from Sigma Chemical Co., St Louis, U.S.A., glucagon from Novo, Copenhagen, Denmark, and adrenaline bitartrate from Rhone Poulenc, Paris, France. Collagenase (CLS II) was from Worthington Biochemical Corp., Freehold, N.J., U.S.A., and Dulbecco's modified Eagle's medium (powder) from Gibco, Grand Island, N.Y., U.S.A.

Stock solutions of DTIC (80 mM) were prepared by dissolving it in 100mM HCl immediately before the incubations. Pure DTIC

* To whom correspondence should be addressed.

and the stock solutions were kept protected from light.

*Assay of cAMP phosphodiesterase.*—Adult male Wistar rat liver was homogenized with a Potter–Elvehjem glass-teflon homogenizer in a buffer containing 100mM tris-HCl (pH 7·5) and 4mM $MgCl_2$. The homogenate was centrifuged at 2000 *g* for 10 min, and the supernatant was treated with $(NH_4)_2SO_4$ to give 55%. The fraction precipitated by $(NH_4)_2SO_4$ was dissolved by dialysing it overnight with two changes against the tris buffer, and stored in aliquots at −80°C. Unless otherwise stated, this preparation was used as the enzyme source in the phosphodiesterase reactions.

The phosphodiesterase assay was carried out essentially as described previously (Christoffersen *et al.*, 1973). The reaction was run at 30°C in a final volume of 400 μl in 100mM tris-HCl (pH 7·5) and 4mM $MgCl_2$. The enzyme activity was measured at various times at various concentrations of cAMP, with [$^3$H]cAMP (∼10,000 ct/incubate). The amount of enzyme per incubate was varied between 75 and 1500 μg protein, according to the substrate concentration. The reaction was terminated by heating at 95°C for 2 min. [$^{14}$C]cAMP (∼5000 ct/min/tube) was added for recovery determination. The cAMP remaining after the incubation was isolated by paper chromatography as previously described (Christoffersen *et al.*, 1973) and counted by liquid scintillation. In kinetic analyses, estimation of initial reaction velocity was based on several incubation times and extrapolating to zero.

*Cells used.*—Previously described procedures were used for hepatocyte isolation (Berg *et al.*, 1972; Seglen, 1972; Christoffersen & Berg, 1974) and incubation (Christoffersen & Berg, 1975). Cell viability, determined by trypan-blue exclusion, was 95–97%. The incubation buffer contained: 119·0mM NaCl, 3·0mM KCl, 2·0mM $CaCl_2$, 1·2mM $MgSO_4$, 2·4mM $KH_2PO_4$, 24·9mM $NaHCO_3$, with 10mM glucose (pH = 7·4). The reaction was terminated by addition of trichloroacetic acid (3·3% final).

$MH_1C_1$ hepatoma cells (Richardson *et al.*, 1969) were obtained from the American Type Culture Collection, Rockville, Md, U.S.A. The cells were cultured as incomplete monolayers in Falcon plastic flasks (75 cm$^2$), in 10ml Dulbecco's modified Eagle's medium, supplemented with 10% horse serum, 2·5% foetal calf serum, penicillin (100 u/ml), streptomycin (0·1 mg/ml) and nystatin (60 u/ml). Medium change was at 3–4 days and subculturing at 7 days. The cells were used for experiments 7 days after seeding, when they were nearly fully grown. Incubations were done in the culture medium described above, and the reactions were stopped by addition of 5% trichloroacetic acid, after rapid removal of medium and washing (twice) of the cell layers with saline.

*Determination of cAMP.*—The samples were centrifuged, and the trichloroacetic acid in the supernatants was neutralized with $CaCO_3$, as described by Tihon *et al.* (1977). cAMP was measured by a radioimmunoassay (Steiner *et al.*, 1969) using acetylation of the samples (Harper & Brooker, 1975; Frandsen & Krishna, 1976) and acetylated [$^3$H]cAMP as ligand (Skomedal *et al.*, 1977).

*Other procedures.*—Protein was determined by the method of Lowry. Cells were counted with a Bürker haemacytometer.

### RESULTS

### *Effects on phosphodiesterase*

As shown in Table I, DTIC inhibited the breakdown of cAMP in a rat liver 40,000*g* supernatant, when a low concentration (0·25 μM) of substrate was used to allow measurement of the low-$K_m$ form(s) of the cAMP phosphodiesterase. DTIC did not seem to affect the high-$K_m$ form, as no inhibition was seen with the use of 2mM cAMP as substrate (Table I).

DTIC inhibited the low-$K_m$ phospho-

TABLE I.—*Effect of DTIC on cAMP phosphodiesterase activity in rat-liver supernatant**

| | nmol cyclic AMP hydrolysed/mg protein/20 min | |
|---|---|---|
| | High substrate conc.† | Low substrate conc.‡ |
| No addition | 182·7 ± 8·8 | 0·941 ± 0·020 |
| DTIC, 1 mM | 182·1 ± 10·2 | 0·688 ± 0·019 |
| DTIC, 2 mM | 173·6 ± 11·6 | 0·461 ± 0·033 |

* A 40,000*g* supernatant was used. The values represent mean ± s.e. of 3–5 determinations.

† Initial conc. 2mM cAMP; 520 μg protein per incubate.

‡ Initial conc. 0·25μM cAMP; 52 μg protein per incubate.

FIG. 1.—Double reciprocal plot of the cAMP phosphodiesterase activity of an ammonium sulphate-precipitable fraction of the 2000*g* supernatant (see Methods) from rat liver, measured at substrate concentration between 0·073 and 10·5 $\mu$M.

diesterase in various preparations of rat liver (data not shown). Routinely, an ammonium-sulphate-precipitated 2000*g* supernatant, prepared as described above, was used. The hydrolysis of cAMP in that preparation is shown as a double reciprocal plot in Fig. 1. Using substrate concentrations in the low range (50nM–10$\mu$M), the data indicated two components of enzyme activity, with $K_m$ at about 0·6 $\mu$M and 2·5 $\mu$M.

Some characteristics of the inhibition by DTIC are given in Fig. 2 A–C. Fig. 2B shows dose-response curves for DTIC and theophylline, using 0·25 $\mu$M of substrate. 50% inhibition was obtained at 790 $\mu$M for DTIC and at 350 $\mu$M for theophylline. The inhibition by DTIC apparently did not require a preincubation period, as it was evident from the beginning of the incubation (Fig. 2A). This differs from the phosphodiesterase inhibition produced by the alkylating agent chlorambucil, which involves a progressive time-dependent change of the enzyme in presence of the drug (Tisdale, 1974). A number of experiments under various conditions indicated that the inhibition by DTIC was almost entirely of the competitive kind. Hoftsee

FIG. 2.—Inhibitory effect of DTIC on cAMP phosphodiesterase activity in an ammonium sulphate-precipitable fraction of the 2000*g* supernatant of rat liver. A: Time course of the phosphodiesterase reaction in the absence (—○—) or presence (—●—) of 2mM DTIC, at 0·25$\mu$M cAMP as initial substrate concentration. B: Dose-response relationship for the effect of DTIC (●) on the phosphodiesterase activity, with theophylline (▲) for comparison. The comparison of activities was based on 5min incubations. Substrate concentration 0·25$\mu$M. C: Hofstee plot of the inhibition by DTIC of the phosphodiesterase activity, measured at substrate concentration 0·25$\mu$M, without (a), or with 0·5mM (b) or 2mM (c), DTIC.

plots of one experiment are given in Fig. 2C.

DTIC was protected from light until added to the reaction vials, but the incubations were not run in the dark. It is unlikely that extensive photodecomposition will occur within the time-span of incubation (Beal *et al.*, 1976), and the inhibition was immediately manifest. Control experiments showed that DTIC exposed to sunlight or UV for 1 h inhibited the phosphodiesterase essentially in the same way as in the standard experiments.

*Effects on intact cells*

To test whether DTIC also influences the phosphodiesterase in intact cells, we investigated its effect on cAMP accumulation in response to hormones in primary hepatocyte suspensions and $MH_1C_1$ hepatoma cell monolayers in culture (Table II).

In hepatocyte suspensions, the presence of 2 mM of either DTIC or theophylline approximately doubled the increase of cAMP produced by a supramaximal concentration (1·4 μM) of glucagon. The cells were preincubated with the phosphodiesterase inhibitors for 10 min, followed by exposure to glucagon for 60 sec. When the cells were incubated with DTIC or theophylline alone (*i.e.* without glucagon), only marginal increases in the cAMP levels were seen.

Similarly, in $MH_1C_1$ hepatoma cells, pretreatment (10 min) with 2mM DTIC or theophylline led to a 2–3-fold amplification of the cAMP response to adrenaline (50 μM, 60sec exposure). In these experiments the effect of DTIC or theophylline alone on cAMP levels (*i.e.* without subsequent adrenaline exposure) was not examined in detail, but later studies (Haffner & Christoffersen, unpublished) have shown significantly raised cAMP in $MH_1C_1$ cells after DTIC or other phosphodiesterase inhibitors.

TABLE II.—*Effect of DTIC and theophylline on basal and hormone-stimulated cAMP levels in rat hepatocytes and $MH_1C_1$ hepatoma cells**

| Additions | pmol cAMP/mg protein: Hepatocytes† | $MH_1C_1$‡ |
|---|---|---|
| None | 2·1 ± 0·8 | 0·8 ± 0·2 |
| DTIC (2 mM) | 2·6 ± 0·3 | 1·2 ± 0·3 |
| Theophylline (2 mM) | 2·7 ± 0·5 | 1·1 ± 0·2 |
| Glucagon (1·4 μM) | 20·4 ± 3·9 | — |
| Glucagon + DTIC | 44·5 ± 9·5 | — |
| Glucagon + theophylline | 43·6 ± 9·2 | — |
| Adrenaline (50 μM) | — | 2·7 ± 0·3 |
| Adrenaline + DTIC | — | 7·3 ± 0·3 |
| Adrenaline + theophylline | — | 7·0 ± 1·0 |

* The values given are cAMP levels after 10 min with or without DTIC or theophylline, followed by 1min exposure to hormone. Mean ± s.e. of determinations in 5 experiments on each cell type. Note that the results from the two kinds of cells are not directly comparable because of different incubation conditions.

† Incubated as suspensions in Krebs–Ringer bicarbonate buffer.

‡ Incubated as monolayers in Dulbecco–Eagle medium.

### DISCUSSION

These results show that the antitumour agent DTIC is a competitive inhibitor of the low-$K_m$ form of cAMP phosphodiesterase, and the ability of DTIC to amplify hormone effects on cAMP accumulation in hepatocytes and hepatoma cells indicates that the inhibition of the phosphodiesterase is also manifested in intact cells. Since about 800 μM was necessary to achieve 50% inhibition of the phosphodiesterase, DTIC is apparently not a particularly potent inhibitor. However, its potency was of about the same order as that of the classical (though not very strong) phosphodiesterase inhibitor theophylline, both as inhibitor in the cell-free phosphodiesterase assay and in augmenting the cAMP response to glucagon and adrenaline in intact cells.

It is not clear whether this phosphodiesterase inhibition plays any role in the antitumour and growth-inhibitory effects of DTIC. The mechanism of action of this drug remains obscure, despite much study (Loo, 1975; Loo *et al.*, 1976; Bono, 1976; Beal *et al.*, 1976). Several theories have been proposed, including purine antimetabolite action (Loo *et al.*, 1968), release of an alkylating methyl radical

(Skibba *et al.*, 1970; Gerulath & Loo, 1972) and interaction with SH-groups (Yamamoto, 1969). However, none of these hypotheses have so far gained definitive support. The present study of the effects of DTIC on phosphodiesterase and cAMP in liver was provoked by the growth-inhibitory and differentiating effect of cAMP on many cell types (Pastan *et al.*, 1975; Friedman, 1976), and by the fact that several other imidazole and imidazolidinone derivatives have been shown to be cAMP phosphodiesterase inhibitors (Chasin & Harris, 1976). DTIC inhibits cells in $G_1$ as well as $G_2$ (Bono, 1976; Gerulath *et al.*, 1974), which could be compatible with a cAMP-mediated effect (Friedman *et al.*, 1976). Furthermore, in cultured mouse neuroblastoma cells, treatment with DTIC (10 $\mu$g/ml) causes increased activity of tyrosine hydroxylase, choline acetyltransferase and acetylcholinesterase (Culver *et al.*, 1977). These effects of DTIC, which may be considered as manifestations of biochemical differentiation of the neuroblastoma cells, are also seen after dibutyryl cAMP administration (Prasad, 1975). However, the increase of these enzyme activities after DTIC were not accompanied by any demonstrable increase in cAMP level or inhibition of phosphodiesterase activity in the study by Culver *et al.* (1977) with 10 $\mu$g/ml of DTIC. With the concentrations of DTIC used here, the phosphodiesterase was inhibited. Further studies (Haffner & Christoffersen, unpublished) have shown potentiation of cAMP responses to adrenaline in $MH_1C_1$ cells by DTIC at 20 $\mu$g/ml.

There is some evidence that certain metabolites of DTIC may be responsible for the growth-inhibitory effects of the drug. DTIC is partly demethylated *in vivo* to yield the monomethyl derivative (MTIC; Skibba *et al.*, 1970), which is also growth-inhibitory, although it is unclear whether the amount formed is sufficient to account for the effects of DTIC (Beal *et al.*, 1976). The role of other degradation products formed by photodecomposition and possibly also *in vivo* has been discussed (Loo, 1975; Loo *et al.*, 1976). It will thus be of interest to examine the effect on cAMP metabolism of various metabolites and analogues of DTIC. Further investigations are necessary to clarify whether cAMP is involved in any aspect of the cytotoxic effect of DTIC. Such studies might also contribute to the understanding of the potential in cancer treatment of substances that act on the cAMP system.

This work was supported by the Norwegian Cancer Society (Landsforeningen mot Kreft) and the Norwegian Council for Science and the Humanities.

### REFERENCES

BEAL, D. D., SKIBBA, J. L., WHITNABLE, K. K. & BRYAN, G. T. (1976) Effects of 5-(3,3-dimethyl-1-triazeno)imidazole-4-carboxamide and its metabolites on Novikoff hepatoma cells. *Cancer Res.*, **36**, 2827.

BERG, T., BOMAN, D. & SEGLEN, P. O. (1972) Induction of tryptophan oxygenase in primary rat liver cell suspensions by glucocorticoid hormone. *Exp. Cell Res.*, **72**, 571.

BONO, V. H. (1976) Studies on the mechanism of action of DTIC (NSC-45388). *Cancer Treat. Rep.*, **60**, 141.

CHASIN, M. & HARRIS, D. N. (1976) Inhibitors and activators of cyclic nucleotide phosphodiesterase. In *Advances in Cyclic Nucleotide Research*, Vol. 7. Eds Greengard & Robison. New York: Raven Press. p. 225.

CHRISTOFFERSEN, T., MØRLAND, J., OSNES, J.-B. & ØYE, I. (1973) Development of cyclic AMP metabolism in rat liver: A correlative study of tissue levels of cyclic AMP, accumulation of cyclic AMP in slices, adenylate cyclase activity and cyclic nucleotide phosphodiesterase activity. *Biochim. Biophys. Acta*, **313**, 338.

CHRISTOFFERSEN, T. & BERG, T. (1974) Glucagon control of cyclic AMP accumulation in isolated intact rat liver parenchymal cells *in vitro*. *Biochim. Biophys. Acta*, **338**, 408.

CHRISTOFFERSEN, T. & BERG, T. (1975) Altered hormone control of cyclic AMP formation in isolated parenchymal liver cells from rats treated with 2-acetylaminofluorene. *Biochim. Biophys. Acta*, **381**, 72.

CULVER, B., SAHU, S. K., VERNADAKIS, A. & PRASAD, K. N. (1977) Effects of 5-(3,3-dimethyl-1-triazeno)imidazole-4-carboxamide [NSC 45388, DTIC] on neuroblastoma cells in culture. *Biochem. Biophys. Res. Commun.*, **76**, 778.

FRANDSEN, E. K. & KRISHNA, G. (1976) A simple ultrasensitive method for the assay of cyclic AMP and cyclic GMP in tissues. *Life Sciences*, **18**, 529.

FRIEDMAN, D. L. (1976) Role of cyclic nucleotides in cell growth and differentiation. *Physiol. Rev.*, **56**, 652.

FRIEDMAN, D. L., JOHNSON, R. A. & ZEILIG, C. E. (1976) The role of cyclic nucleotides in the cell cycle. In *Advances in Cyclic Nucleotide Research*, Vol. 7. Eds Greengard & Robison. New York: Raven Press. p. 69.

GERULATH, A. H., BARRANCO, S. C. & HUMPHREY, R. M. (1974) The effects of treatments with 5-(3,3-dimethyl-1-triazeno)imidazole-4-carboxamide in darkness and light on survival and progression in Chinese hamster ovary cells *in vitro*. *Cancer Res.*, **34**, 1921.

GERULATH, A. H. & LOO, T. L. (1972) Mechanism of action of 5-(3,3-dimethyl-1-triazeno)imidazole-4-carboxamide in mammalian cells in culture. *Biochem. Pharmacol.*, **21**, 2335.

HARPER, J. F. & BROOKER, G. (1975) Femtomole sensitive radioimmunoassay for cyclic AMP and cyclic GMP after 2'0-acetylation by acetic anhydride in aqueous solution. *J. Cycl. Nucl. Res.*, **1**, 207.

KOTANI, M., KOIZUMI, Y., YAMADA, T., KAWASAKI, A. & AKABANE, T. (1978) Increase of cyclic adenosine 3':5'-monophosphate concentration in transplantable lymphoma cells by *Vinca* alkaloids. *Cancer Res.*, **38**, 3094.

LOO, T. L. (1975) Triazeno derivatives. In *Antineoplastic and Immunosuppressive Agents, Part II. Handbook of Experimental Pharmacology*, Vol. 38-II. Eds Sartorelli & Johns. Berlin: Springer Verlag. p. 544.

LOO, T. L., HOUSHOLDER, G. E., GERULATH, A. H., SAUNDERS, P. H. & FARQUHAR, D. (1976) Mechanism of action and pharmacology studies with DTIC (NSC-45388). *Cancer Treat. Rep.*, **60**, 149.

LOO, T. L., LUCE, J. K., JARDINE, J. H. & FREI, E., III (1968) Pharmacologic studies of the antitumor agent 5-(dimethyl-triazeno)imidazole-4-carboxamide. *Cancer Res.*, **28**, 2448.

PASTAN, I. H., JOHNSON, G. S. & ANDERSON, W. B. (1975) Role of cyclic nucleotides in growth control. *Annu. Rev. Biochem.*, **44**, 491.

PRASAD, K. N. (1975) Differentiation of neuroblastoma cells in culture. *Biol. Rev.*, **50**, 129.

RICHARDSON, U. I., TASHIJAN, A. H. & LEVINE, L. (1969) Establishment of a clonal strain of hepatoma cells which secrete albumin. *J. Cell. Biol.*, **40**, 236.

RUDOLPH, S. A., GREENGARD, P. & MALAWISTA, S. E. (1977) Effects of colchicine on cyclic AMP levels in human leukocytes. *Proc. Natl Acad. Sci. U.S.A.*, **74**, 3404.

SEGLEN, P. O. (1972) Preparation of rat liver cells. I. Effect of $Ca^{2+}$ on enzymatic dispersion of isolated, perfused liver. *Exp. Cell Res.*, **74**, 450.

SKIBBA, J. L., BEAL, D. D., RAMIREZ, G. & BRYAN, G. T. (1970) N-Demethylation of the antineoplastic agent 4(5)-(3,3-dimethyl-1-triazeno)imidazole5(4)carboxamide by rats and man. *Cancer Res.*, **30**, 147.

SKOMEDAL, T., OSNES, J.-B., GRYNNE, B., SJETNAN, A. E. & ØYE, I. (1977) A new radioimmunoassay for cyclic AMP obtained by acetylation of both $^{3}H$-cyclic AMP and unlabelled cyclic AMP. Abstract. 3rd Scand. Symp. Cyclic Nucl., University of Linköping.

STEINER, A. L., KIPNIS, D. M., UTIGER, R. & PARKER, C. W. (1969) Radioimmunoassay for the measurement of adenosine 3',5'-cyclic phosphate. *Proc. Natl Acad. Sci. U.S.A.*, **64**, 367.

TIHON, C., GOREN, M. B., SPITZ, E. & RICKENBERG, H. V. (1977) Convenient elimination of trichloroacetic acid prior to radioimmunoassay of cyclic nucleotides. *Analyt. Biochem.*, **80**, 652.

TISDALE, M. J. (1974) The reaction of alkylating agents with cyclic 3',5'-nucleotide phosphodiesterase. *Chem. Biol. Interact.*, **9**, 145.

TISDALE, M. J. & PHILLIPS, B. J. (1975*a*) Inhibition of cyclic 3',5'-nucleotide phosphodiesterase—a possible mechanism of action of bifunctional alkylating agents. *Biochem. Pharmacol.*, **24**, 205.

TISDALE, M. J. & PHILLIPS, B. J. (1975*b*) Comparative effects of other anti-tumour agents on the intracellular level of adenosine 3'5'-monophosphate in Walker carcinoma. *Biochem. Pharmacol.*, **24**, 1271.

YAMAMOTO, I. (1969) 4(or 5)-Diazoimidazole-5(or 4) carboxamide and related triazeno-imidazoles as antibacterial agents: their effects on nucleic acid metabolism of *Escherichia coli B*. *Biochem. Pharmacol.*, **18**, 1463.